# Efficacy and safety of the combination of modern medicine and traditional Chinese medicine in pulmonary fibrosis caused by novel coronavirus disease: A protocol for Bayesian network meta-analysis

**DOI:** 10.1097/MD.0000000000028282

**Published:** 2021-12-23

**Authors:** Feiran Li, Guanyu Wang, Wei Zhang, Caiqing Zhang

**Affiliations:** a First College of Clinical Medicine, Shandong University of Traditional Chinese Medicine, Jinan, China; b Department of Health Care, Huaiyin People's Hospital, Jinan City, Shandong Province, China; c Affiliated Hospital of Shandong University of Traditional Chinese, Medicine, Jinan, China; d Pulmonary and Critical Care Medicine, Shandong Province's Second General Hospital (Shandong Province ENT Hospital), Shandong University of Traditional Chinese Medicine, Jinan, China.

**Keywords:** novel coronavirus disease, modern medicine, network meta-analysis, pulmonary fibrosis, traditional Chinese medicine

## Abstract

**Background:**

Novel coronavirus disease (COVID-19) is a kind of pulmonary inflammation induced by New Coronavirus. It seriously threatens people's health and safety. Clinical studies have found that some patients have different degrees of inflammation after discharge from hospital, especially in patients with severe inflammatory lung fibrosis. Early combination of Chinese medicine and modern medicine has important clinical significance. There are still many deficiencies in the current research. We studied the effectiveness of the combination of traditional Chinese medicine and modern medicine in the treatment of pulmonary fibrosis caused by COVID-19, and proposed a network meta-analysis (NMA) scheme.

**Methods:**

According to the search strategy, we will search Chinese and English databases to collect all randomized controlled trials of traditional Chinese medicine combined with modern drugs or only using traditional Chinese medicine for new coronavirus-19-induced pulmonary fibrosis between December 1, 2019 and November 15, 2021. First, the literature was screened according to the eligibility criteria, endnotex9 was used to manage the literature, and the Cochrane Collaboration's tool was used to assess the quality of the included literature. Revman 5.3, Stata 14.2, and gemtc14.3 meta-analysis software was then used for data processing and analysis, and the grading of recommendations assessment will be used to develop and evaluate a hierarchy for classifying the quality of evidence for NMA.

**Results:**

Through the analysis, the ranking of efficacy and safety of various treatments for pulmonary fibrosis caused by COVID-19 will be drawn, thus providing stronger evidence support for the choice of clinical treatment methods.

**Conclusion:**

Traditional Chinese medicine (TCM) combined with modern drugs has played a positive role in the treatment of pulmonary fibrosis caused by COVID-19, and this study may provide more references for the clinical medication of pulmonary fibrosis caused by COVID-19.

**INPLASY registration number:**

INPLASY2021110061.

## 1 Introduction

Since December 2019, novel coronavirus disease (COVID-19) has been discovered in Wuhan and spread rapidly all over the world, and more than 200 million confirmed cases of new coronal pneumonia have been accumulated worldwide, resulting in more than 5,000,000 deaths. The COVID-19 epidemic has become a global phenomenon that seriously threatens people's health and safety, and the world is still in the phase of the COVID-19 pandemic,^[1–3]^ and clinical studies have found that some patients have different degrees of postinflammatory pulmonary fibrosis at discharge, and that postinflammatory pulmonary fibrosis is particularly significant in severe patients, and that COVID-19 can cause pulmonary fibrosis in patients,^[4–6]^ This complication, which seriously threatens the prognosis, also deserves our attention.

Pulmonary fibrosis is a pathological consequence of pulmonary fibrosis as an acute and chronic interstitial lung disease.^[7]^ Pulmonary fibrosis can result from many causes, including age, smoking, viral infections, drug exposure, and genetic predisposition, among others. Consequently, alveolar damage, failure of alveolar epithelial remodeling, proliferation of fibroblasts, excessive deposition of large amounts of extracellular matrix, and destruction of lung tissue architecture occur. Progression of pulmonary fibrosis leads to widening of the interstitial matrix, ultimately compressing and destroying normal lung parenchyma, which damages capillaries, resulting in respiratory failure. Patients with pulmonary fibrosis can present with dyspnea and dry cough after varying degrees of activity. Different degrees of grid shadow can be seen on chest CT images. Pulmonary function is characterized by restrictive ventilation dysfunction and reduced diffusion function, which is one of the important reasons for the decline of patients’ quality of life and disability.^[8]^

COVID-19 is a completely new disease, and despite the controlled epidemic, we know little about its disease behavior and are more inexperienced about the mechanisms by which COVID-19 leads to pulmonary fibrosis. COVID-19 as a kind of viral infection, especially the elderly population is prone to virus induced fibrosis due to immunosenescence and viral infection as cofactors.^[9]^ Chronic inflammation is an important cause of pulmonary fibrosis, COVID-19 inflammation late or convalescent phase, with the improvement of the condition, pulmonary inflammation gradually reduces, however, due to the necrosis and shedding of a large number of alveolar epithelial cells in the previous stage, the body initiates damage repair mechanisms, active my fibroblast foci formation, and thus pulmonary fibrosis.

For the treatment of pulmonary fibrosis, glucocorticoids, antacid preparations, and immunosuppressive agents are commonly used in Western Medicine,^[10]^ and so far, only pirfenidone and nintedanib have been approved by FDA, but their efficacy is not ideal and they have some side effects.^[11]^ In recent years, traditional Chinese medicine (TCM) has made great progress in the treatment of pulmonary fibrosis, to some extent making up for the shortage of Western medical treatment.^[12]^ It can be symptomatic in patients according to different population constitution, different disease course, and according to different geographical, climate, environment and other factors, it has the advantage of individualization, its efficacy is significant, side effects are small, and the mechanism of action involves several pathways and targets, which has shown significant advantages in the battle against COVID-19, effectively blocking disease progression, Promote patient recovery.^[13]^ The treatment protocol of COVID-19 was also introduced in the new diagnosis and treatment protocol for coronavirus pneumonia (Trial VI Edition), in which the total effective rate of Qingfei detoxification decoction was 97%. There are clinical and experimental evidences that many TCMs have antifibrotic potential, and their efficacy is mainly for toning Qi, invigorating blood and promoting water infiltration and dampness, and the mechanisms of their effects mostly involve regulating inflammation, oxidative stress, hydroxyproline, fibrotic signaling pathways, etc, which provide an important strategy for the treatment of pulmonary fibrosis.^[14–16]^

Therefore, early adoption of combined traditional Chinese and modern medicine is of great clinical importance, both for the recovery of lung function and the prognosis of COVID-19 in patients with pulmonary fibrosis, but based on the many shortcomings of the current study, we investigated the effectiveness of combination of traditional Chinese and modern medicine treatment of pulmonary fibrosis caused by COVID-19 and proposed network meta-analysis (NMA) Scheme.

## 2 Methods

In this study, we will use this research will adopt Bayesian network meta-analysis, and NMA. And reported literature review according to the PRISMA 2020 (preferred reporting items for systematic reviews and meta-analyses) PRISMA statement.^[17]^

### 2.1 Study registration

The protocol of this NMA was registered on the international platform for the registration of systematic review and meta-analysis protocols (inplasy). Registration Number is INPLASY2021110061 (URL: https://inplasy.com/inplasy-2021–11-0061/).

### 2.2 Inclusion criteria

#### 2.2.1 Type of study

Explore we will include relevant randomized controlled trials published in China and internationally related to modern medical treatment of pulmonary fibrosis due to COVID-19 in combination with conventional therapies in traditional Chinese medicine. The language is limited to Chinese and English.

#### 2.2.2 Participants

Patients diagnosed with pulmonary fibrosis due to COVID-19. Patients should be in convalescence on COVID-19 and have fibrotic pulmonary manifestations, fulfilling the diagnostic criteria for pulmonary fibrosis. Age, sex, race, or, nationality were not considered.

#### 2.2.3 Interventions

Patients will be randomized into a treatment or control group. The experimental group will receive treatment with conventional modern therapies combined with traditional Chinese medicine plus traditional Chinese medicine (e.g., Lian Hua Qing distemper granules and, Qingfei detoxification decoction, etc) or with traditional Chinese medicine alone, whereas the control group will receive placebo with conventional modern medicine or conventional modern medicine. Conventional modern drugs mainly include pirfenidone, nintedanib, N-acetylcysteine and so on. There are no restrictions on dosage, usage and course.

#### 2.2.4 Control

The control group will receive placebo for conventional modern medicines or conventional modern medicines. Conventional modern for example, drugs mainly include pirfenidone, nintedanib, and N-acetylcysteine, among others. There were no restrictions on dosage, usage, and course of treatment in the intervention and control groups.

#### 2.2.5 Results

Pulmonary function indices, oxygen saturation, and quality of life. Lung function parameters mainly include vital capacity (VC), total lung capacity (TLC), FVC, flv, etc, And will undergo a 6-minutes’ walk distance test according to the American Thoracic Society (ATS) standards to assess pulmonary function assessment will also be included. Will pass arterial oxygen saturation (SaO_2_) and partial pressure of oxygen (PaO_2_) will be used to monitor oxygen saturation. Quality of life will be assessed using the SF-36 form, SCL-90 form, SAS form, and SDS form.

### 2.3 Exclusion criteria

Animal experiments and other studies, for example, non randomized controlled trials, studies without clear criteria for efficacy assessment, reviews, poorly designed studies, cross-sectional studies, duplicates, or plagiarism.

### 2.4 Search strategy

We will comprehensively search the following databases from December 2019 to November 2021: PubMed, China National Knowledge Infrastructure (CNKI), Wanfang database, Cochrane Library, VIP database, Chinese biomedical literature database (sinomed), EMBASE, web of science, and Cochrane controlled trials Central Register of clinical trials, and. Government clinical registration system. Limited to reports published in Chinese and English. The language is limited to Chinese and English. Retrieval skills and considerations will be studied in detail, and the final retrieval strategy will be determined after multiple searches. The search strategy will be constructed in the form of medical subject headings (mesh) combined with synonyms, and combined with keywords, including COVID-19, SASR-cov-2, pulmonary fibrosis, modern medicine, traditional Chinese medicine, clearing lung detoxification, Huoxiang Zhengqi, pulmonary function, randomized controlled trial, and so on. We will investigate the search strategy in detail with professional librarians to determine the most comprehensive final search strategy possible after multiple pre searches. In addition, we manually browsed references of included studies and relevant reviews to identify additional studies. Database search PubMed was used as an example, and the search strategy is shown in Table 1.

**Table 1:** Search strategy for PubMed.

NO.	Search item
#1	“novel coronavirus disease” [MeSH Terms]
#2	“novel coronavirus disease” [Title/Abstract] OR ”Novel Coronavirus 2019” [Title/Abstract] OR ”2019 novel coronaviruspneumonia” [Title/Abstract] OR ”COVID-19” [Title/Abstract] OR “SARS-CoV-2” [Title/Abstract] OR” severe acute respiratory syndrome coronavirus” [Title/Abstract]
#3	#1 OR #2
#4	“pulmonary fibrosis” [MeSH Terms]
#5	“pulmonary fibrosis” [Title/Abstract] OR ” idiopathic interstitial pneumonia” [Title/Abstract] OR ”idiopathic pulmonary fibrosis” [Title/Abstract] OR ”interstitial lung disease” [Title/Abstract] OR ”Lung fibrosis “[Title/Abstract]
#6	#4 OR #5
#7	Medicine, Chinese Traditional[MeSH Terms]
#8	Medicine,Chinese Traditional [Title/Abstract] OR Traditional Chinese Medicine [Title/Abstract] OR Traditional Medicine, Chinese [Title/Abstract] OR Zhong YiXue [Title/Abstract] OR Chinese Traditional Medicine [Title/Abstract] OR Chinese Medicine, Traditional [Title/Abstract] OR Traditional Tongue Diagnos∗[Title/Abstract] OR Tongue Diagnos∗ Traditional [Title/Abstract] OR Traditional Tongue Assessment∗ [Title/Abstract] OR Tongue Assessment, Traditional [Title/Abstract]
#9	#7 OR #8
#10	Lianhua qingwen [Title/Abstract] OR qingwen baidu [Title/Abstract] OR qingfei paidu [Title/Abstract] OR feiyan yihao[Title/Abstract] OR huashi paidu [Title/Abstract] OR huoxiang zhengqi [Title/Abstract] OR yinqiao san [Title/Abstract]
#11	#9 OR #10
#12	Randomized controlled [Publication Type] OR Controlled Clinical Trial [Publication Type] OR Randomized [Title/Abstract] OR Randomly [Title/Abstract] OR random allocation [Title/Abstract]
#13	#3 AND #6 AND #11 AND #12

### 2.5 Study selection and data extraction

In this study, we will use endnotex9 for curation of the literature. All recorded research findings from the above databases and retrieved, manually retrieved studies were imported into endnotex9 for classification management according to a predetermined search strategy. First, 2 independent researchers will browse the initial screening based on the titles and abstracts of the included literatures, then read the full text of the remaining literatures according to the principle of qualifying (eligibility) criteria designed before, and finally identify the suitable eligible (eligible) literatures. And extracted data were used, and the data were recorded in Microsoft excel 2019 software for the recording of extracted data. If the opinions of 2 researchers were not in agreement, they were discussed to reach an agreement, and a third researcher was involved to solve this problem if necessary. The required information including the following data will be extracted: title, country, journal, first author, time of publication, study design, number of participants and demographic characteristics (age, gender, etc), disease course, status, inclusion criteria, exclusion criteria, treatment measures, main study indicators and outcomes. If the relevant data are incomplete, we will attempt to e-mail the authors to obtain the relevant data information. If relevant data remain unavailable, we will only analyze available data and explain the possible impact of missing data.

### 2.6 Risk of bias assessment quality

Will be assessed independently and separately by 2 authors (LFR and WGY) workers according to the Cochrane Collaboration risk of bias tool. In the present study, 2 investigators (LFR and WGY) will perform the risk of bias assessment tool as recommended in the Cochrane Handbook for systematic reviewers 5.3 the quality of the included literature was assessed separately. Where disagreement exists between the 2 researchers, a corresponding decision will be made through discussion. When necessary, a third researcher (zcq or ZW) will make the decision and explain the reason. The following aspects will be used as evaluation criteria: proper application of randomization, application of allocation concealment, blinding of participants and researchers, completeness of results and data, selective reporting of outcomes, and other relevant biases. According to the above criteria, the risk of bias in studies was classified into three levels: “low risk of bias,” “high risk of bias,” and “ambiguous risk of bias.”

### 2.7 Heterogeneity test

Heterogeneity across studies was assessed using Cochran's Q-test and Higgins’ *I*^2^ statistics Tests, and the heterogeneity size as judged by the statistics was assessed. Low heterogeneity was judged when *I*^2^ < 50% and *P* > .10, and high heterogeneity when*I*^2^ ≥ 50% or *P* ≥ .10. A fixed effects model was adopted to combine the statistics in case of low heterogeneity, otherwise a random effects model was adopted for the effect analysis, and the possible reasons for the heterogeneity generation were explored by subgroup analysis and sensitivity analysis.

### 2.8 Statistical analysis

A risk bias plot and heterogeneity test were performed using Review Manager 5.3. The odds ratio (or) was used for dichotomous variables and the mean difference (MD) for continuous variables, and the 95% confidence interval (CI) was used for all interval estimates. Subgroup analysis was performed based on the type of intervention according to clinical heterogeneity. The “network meta” command in stata14.0 software was adopted to draw the network diagram of interventions. A Bayesian network meta-analysis was conducted with gemtc14.3 software to conduct direct evidence and indirect evidence comparisons of each intervention among the included studies. Bayesian inference was performed by fitting the consistency model using Markov chain Monte Carlo, and potential scale reduction factor (psrf) parameters were evaluated in gemtc14.3 software if the psrf value was ≥1.2 indicating that the number of current simulations was insufficient to reach good convergence and the number of simulations was increased for re-evaluation; When the psrf values were in the range of 1.00 to 1.05, which indicated that the convergence of the iteration effect was good, and the closer to 1 indicated that the data had good convergence, a consistency model was used for net meta-analysis. Inconsistency testing was performed when a closed loop existed among interventions, and inconsistency testing was performed using a point split model. If *P* > .05, the consistency model was used for analysis; Otherwise, non concordant type was adopted for analysis. The probability ranking of the efficacy of each intervention was calculated based on the Bayesian analysis model in the gemtc14.3 software, inferring the case of the probability that each measure was the most effective treatment. Subgroup analysis was performed during data analysis based on relevant primary examination indicators, while sensitivity analysis was conducted to assess outcome stability based on the methodological quality of included literatures and relevant primary examination indicators. Finally a comparison–corrected funnel plot was drawn with stata14.0 to identify the presence of small sample effects in relevant outcomes, as well as to assess publication bias.

### 2.9 Quality of evidence assessment

We will assess the quality of evidence using the grading of recommendations assessment development and evaluation (grade) tool in 4 levels: high, moderate, low, and very low.^[18]^

### 2.10 Ethics and dissemination

Since the data of this study were all from the literature and did not involve any personal privacy, no ethical approval was required. The results of this study will be presented in a peer journal or at a conference.

## 3 Discussion

COVID-19 has become a major global burden. As a new disease, there is no precedent in the treatment of substantive abnormalities of the lung and pulmonary fibrosis. We can only find common characteristics from similar diseases, carry out basic research and clinical trials, and finally make COVID-19 patients get better treatment.COVID-19, an acute viral infectious disease caused by sars-cov-2, has a short disease course, and most patients with mild disease do not develop pulmonary fibrosis, but a high proportion of severe and critically ill patients develop pulmonary fibrosis, which seriously threatens the life safety of patients.^[19–22]^ For pulmonary fibrosis due to COVID-19, therapeutic options are quite limited. In modern medicine, drugs such as pirfenidone and nintedanib have become one of the most promising drugs against pulmonary fibrosis, and although they can slow the progression of pulmonary fibrosis and improve quality of life, they cannot cure pulmonary fibrosis fundamentally and have some side effects. TCM has accumulated extensive experience in treating diseases with a long history, and TCM can improve clinical symptoms, control disease progression, shorten treatment time, reduce hormone dosage, and alleviate complications, which provides us with a great potential to discover new drugs. TCM focuses on the holistic view, and the therapeutic ideas are “Fuzheng to expel evil,” in order to promote immunity mainly, supplemented by symptomatic treatment. “syndrome differentiation and treatment” is the basic principle of TCM to recognize and treat diseases, and to combine the characteristics of patient's body and condition as well as climate to make individualized treatment, which is the advantage of TCM.^[23–25]^ A new therapeutic approach is available through the model of combining traditional Chinese medicine and modern medicine and may be effective in improving lung function, improving their quality of life, and prolonging life in patients with pulmonary fibrosis caused by COVID-19. TCM can be used as a complementary therapy in modern drug therapy, and play a synergistic role of both together. Therefore, we will use the NMA method to compare the advantages and disadvantages of various treatments, evaluate the safety and efficacy of TCM in the treatment of pulmonary fibrosis caused by COVID-19 by designing this study to rank different treatments, and thus provide a basis for clinicians and patients. Although the advantages of network meta-analysis are numerous, our study still inevitably has some limitations and deficiencies, such as the data are derived from references rather than direct original data, and there is a language bias, the analysis results can be difficult to bias. In addition, the mechanism of TCM in preventing and treating pulmonary fibrosis is complex, and the mechanism of the interaction between TCM and modern drugs is still unclear. Therefore, we need to have more experimental studies with high methodological quality, large sample size, and longer intervention duration to continuously improve the quality of evidence-based medicine to provide evidence support and guidance for developing a reasonable and effective diagnostic protocol for pulmonary fibrosis caused by COVID-19.

## Author contributions

**Conceptualization:** Feiran Li, Guanyu Wang.

**Data curation:** Feiran Li, Guanyu Wang.

**Formal analysis:** Feiran Li, Guanyu Wang.

**Methodology:** Feiran Li, Guanyu Wang.

**Project administration:** Feiran Li, Guanyu Wang, Caiqing Zhang.

**Software:** Feiran Li, Guanyu Wang.

**Supervision:** Wei Zhang, Caiqing Zhang.

**Writing – original draft:** Feiran Li, Guanyu Wang.

**Writing – review & editing:** Feiran Li, Guanyu Wang.
